# Targeted Sequencing in Chromosome 17q Linkage Region Identifies Familial Glioma Candidates in the Gliogene Consortium

**DOI:** 10.1038/srep08278

**Published:** 2015-02-05

**Authors:** Ali Jalali, E. Susan Amirian, Matthew N. Bainbridge, Georgina N. Armstrong, Yanhong Liu, Spyros Tsavachidis, Shalini N. Jhangiani, Sharon E. Plon, Ching C. Lau, Elizabeth B. Claus, Jill S. Barnholtz-Sloan, Dora Il'yasova, Joellen Schildkraut, Francis Ali-Osman, Siegal Sadetzki, Christoffer Johansen, Richard S. Houlston, Robert B. Jenkins, Daniel Lachance, Sara H. Olson, Jonine L. Bernstein, Ryan T. Merrell, Margaret R. Wrensch, Faith G. Davis, Rose Lai, Sanjay Shete, Kenneth Aldape, Christopher I. Amos, Donna M. Muzny, Richard A. Gibbs, Beatrice S. Melin, Melissa L. Bondy

**Affiliations:** 1 Department of Neurosurgery, Baylor College of Medicine, Houston, Texas; 2 Department of Pediatrics, Division of Hematology-Oncology, Dan L. Duncan Cancer Center, Baylor College of Medicine, Houston, Texas; 3 Codified Genomics, LLC, Houston Texas; 4 Human Genome Sequencing Center, Baylor College of Medicine, Houston, Texas; 5 Department of Epidemiology and Public Health, Yale University School of Medicine, New Haven, Connecticut; 6 Department of Neurosurgery, Brigham and Women's Hospital, Boston, Massachusetts; 7 Case Comprehensive Cancer Center, Case Western Reserve University School of Medicine, Cleveland, Ohio; 8 Department of Epidemiology and Biostatistics, Georgia State University School of Public Health, Atlanta, Georgia; 9 Cancer Control and Prevention Program, Department of Community and Family Medicine, Duke University Medical Center, Durham, North Carolina; 10 Department of Surgery, Duke University Medical Center, Durham, North Carolina; 11 Cancer and Radiation Epidemiology Unit, Gertner Institute, Chaim Sheba Medical Center, Tel Hashomer; 12 Sackler School of Medicine, Tel-Aviv University, Tel-Aviv, Israel; 13 Institute of Cancer Epidemiology, Danish Cancer Society, Copenhagen, Denmark; 14 Rigshospitalet, University of Copenhagen, Copenhagen, Denmark; 15 Section of Cancer Genetics, Institute of Cancer Research, Sutton, Surrey, United Kingdom; 16 Mayo Clinic Comprehensive Cancer Center, Mayo Clinic, Rochester, Minnesota; 17 Department of Epidemiology and Biostatistics, Memorial Sloan-Kettering Cancer Center, New York, New York; 18 Department of Neurology, NorthShore University HealthSystem, Evanston, Illinois; 19 Department of Neurological Surgery, University of California, San Francisco, San Francisco, California; 20 Department of Public Health Services, University of Alberta, Edmonton, Alberta, Canada; 21 Departments of Neurology, Neurosurgery, and Preventive Medicine, The University of Southern California Keck School of Medicine, Los Angeles, California; 22 Department of Biostatistics, The University of Texas MD Anderson Cancer Center, Houston, Texas; 23 Department of Pathology, The University of Texas MD Anderson Cancer Center, Houston, Texas; 24 Department of Community and Family Medicine, Department of Genetics, Norris Cotton Cancer Center, Geisel School of Medicine at Dartmouth; Hanover, New Hampshire; 25 Department of Radiation Sciences Oncology, Umeå University, Umeå, Sweden

## Abstract

Glioma is a rare, but highly fatal, cancer that accounts for the majority of malignant primary brain tumors. Inherited predisposition to glioma has been consistently observed within non-syndromic families. Our previous studies, which involved non-parametric and parametric linkage analyses, both yielded significant linkage peaks on chromosome 17q. Here, we use data from next generation and Sanger sequencing to identify familial glioma candidate genes and variants on chromosome 17q for further investigation. We applied a filtering schema to narrow the original list of 4830 annotated variants down to 21 very rare (<0.1% frequency), non-synonymous variants. Our findings implicate the *MYO19* and *KIF18B* genes and rare variants in *SPAG9* and *RUNDC1* as candidates worthy of further investigation. Burden testing and functional studies are planned.

Gliomas comprise approximately 75% of all malignant primary brain tumors (PBTs) and account for an estimated 4% of cancer deaths in the United States^1^,^2^,^3^,^4^. Glioblastoma multiforme (GBM) is the most common type of glioma, constituting nearly 65% of cases, with an incidence rate of 2–3 per 100,000 in the United States and Europe. The 5-year survival for individuals with GBM is only about 10%, and median survival time is an estimated 12–14 months.

Despite decades of research, there are few established risk factors for glioma^5^. A number of candidate gene and genome wide association studies have been conducted^6^,^7^,^8^,^9^,^10^, and have, thus far, revealed seven low-penetrant susceptibility loci associated with sporadic glioma formation^8^. With regard to familial glioma, known single gene disorders, such as neurofibromatosis, tuberous sclerosis, and Li-Fraumeni and Turcot's syndromes, predispose patients to glioma formation, but cannot explain more than a minute proportion of cases^11^. Therefore, the factors responsible for first-degree relatives of glioma patients having approximately twice the risk of glioma formation compared to unrelated individuals remains unclear^12^.

Because the genetic basis of familial glioma remains enigmatic, the “Genetic Epidemiology of Glioma International Consortium” (Gliogene Consortium) was formed in 2006 to recruit families affected by glioma in 14 different institutions across five countries^11^,^13^,^14^. This consortium has provided an unprecedented opportunity to further our understanding of the heritability of this rare, though highly fatal, condition, with the ultimate goal of uncovering enough information about glioma susceptibility to allow for the screening and genetic counseling of high-risk individuals and families. Our previous studies, which involved non-parametric and parametric linkage analyses, both yielded significant linkage peaks only on chromosome 17q (parametric linkage score: 3.1, nonparametric linkage score: 3.39)^13^,^15^. Because of the concordance between these prior findings, we conducted targeted sequencing focused on this region of chromosome 17q, with the aim of identifying the variant(s) or gene(s) that could explain linkage across this region to familial glioma. We, additionally, characterized deleterious rare variants within this chromosomal region among these glioma families.

## Results

**Figure 1:**
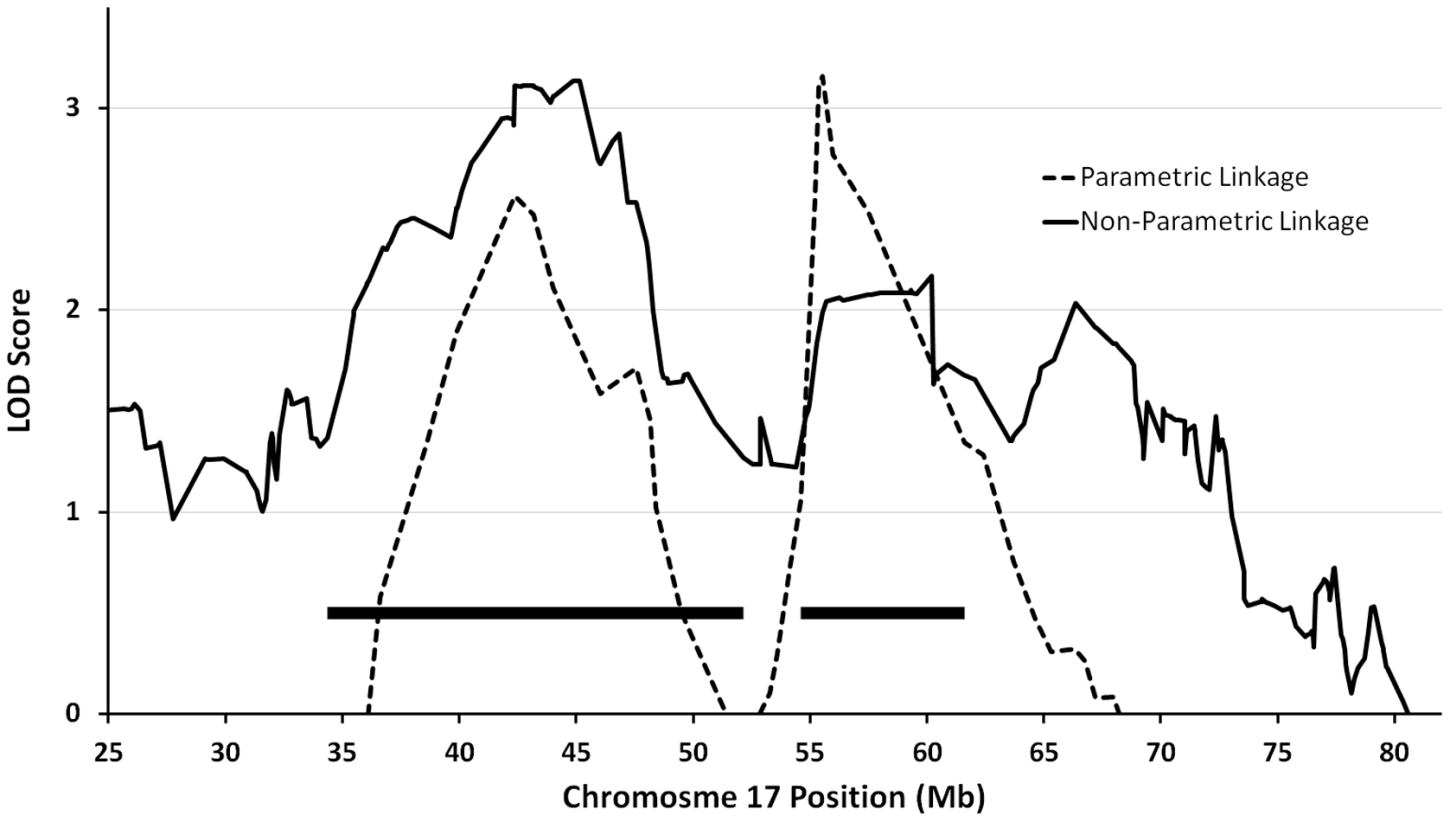
Combined results of parametric and non-parametric linkage analysis on Gliogene families. The only significant peaks are on chromosome 17q. All coordinates are based on human genome version 19 sequence (GRCh37). Solid bars represent the linkage locus, defined as the 1.7 LOD drop region.

The linkage region was defined as the 1.7 LOD drop region from linkage peaks, which spans bases 34,355,567-52,135,011 and 54,612,056-61,596,548 (GRCh37 coordinates) on chromosome 17 (solid bars in Fig. 1).

A total of 203 individuals from 23 families were successfully sequenced (33 affected, 170 unaffected) to an average depth of coverage of 95 fold in the target regions. Of these families, 20 had at least one affected individual sequenced (probands of three families failed sequencing and were subsequently excluded from additional analysis). Information on the demographics of affected individuals, whether sequencing was completed, and the relationship between affected individuals in each family is summarized in Table 1. Affected individuals were not sequenced if they were deceased prior to study initiation, or if a specimen was otherwise unobtainable.

**Figure 2:**
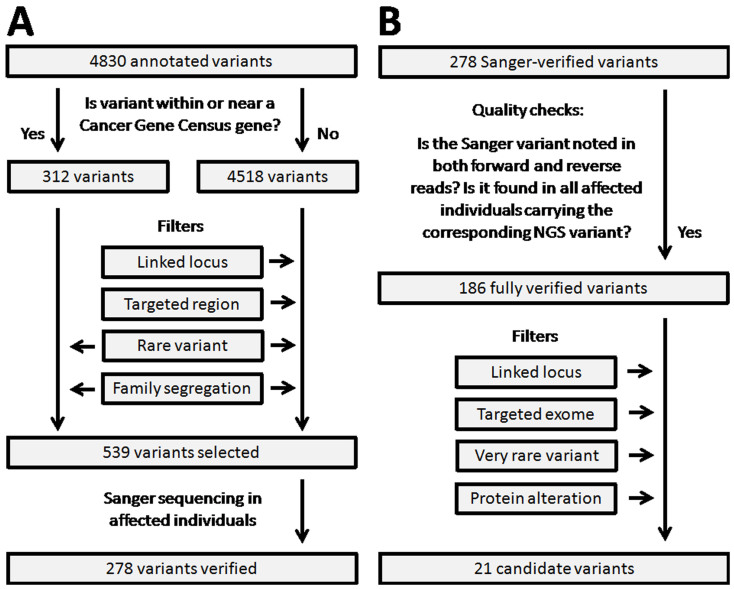
Chromosome 17q variant filtering schema. (a) Flowchart depicts the initial set of variant filters used to narrow the list of variants for Sanger sequencing. “Targeted region” and “Linked locus” filters were waived for **v**ariants in Cancer Gene Census^53^ genes. (b) Flowchart depicts the quality check process for Sanger-confirmed variants and the post Sanger sequencing set of variant filters applied to arrive at the final list of 21 candidate variants.

After alignment and variant calling, a total of 4,830 variants were annotated. After removing common variants, variants mapping outside our selected target regions, variants only present in unaffected individuals, and variants not segregating among affected individuals in the same family, there were 539 remaining variants, which were subsequently submitted for Sanger sequencing verification for the affected individuals (Fig. 2A). All of the variants in our final list (Table 2) were located within the linkage region.

### Sanger sequencing

Out of 539 variants interrogated by Sanger sequencing, some failed initial design and quality checks, including several attempts at successful primer design, in part due to the location of the variants within high-repeat or low-complexity regions or within duplicated regions. A total of 278 variants (51.6%) were validated in at least in a subset of affected individuals (Fig. 2B). Reads not meeting the aforementioned quality control criteria were removed, leaving 186 variants (66.9%) that were fully verified by Sanger sequencing. Our final filtering criteria were then applied (Fig. 2B). A final list of 21 candidate variants, all missense mutations, was obtained (Table 2).

### Candidate variants

The 21 candidate variants on our final list are each private to individual families (Table 2). There were 15 candidate variants present in more than one affected individual per family (“2/2” affected ratio in Table 2), and the remaining six variants were in families with only one sequenced affected individual. Of the 15 shared variants, three variants are novel, never having been reported in a publicly available variant database (dbSNP, ESP, 1000 Genome Project). These include two missense mutations in *RSAD1* and *MYO19* in both affected and 3 out of 8 unaffected members of Family J, and a missense mutation in *G6PC* in both affected and 0 out of 5 unaffected members of Family A.

Although each specific variant on our final list was private to an individual family, there were two genes for which more than one family had variants that made the final list. *MYO19* was implicated in Families J and O, and *KIF18B* was implicated in Families G and B (Table 2).

**Figure 3:**
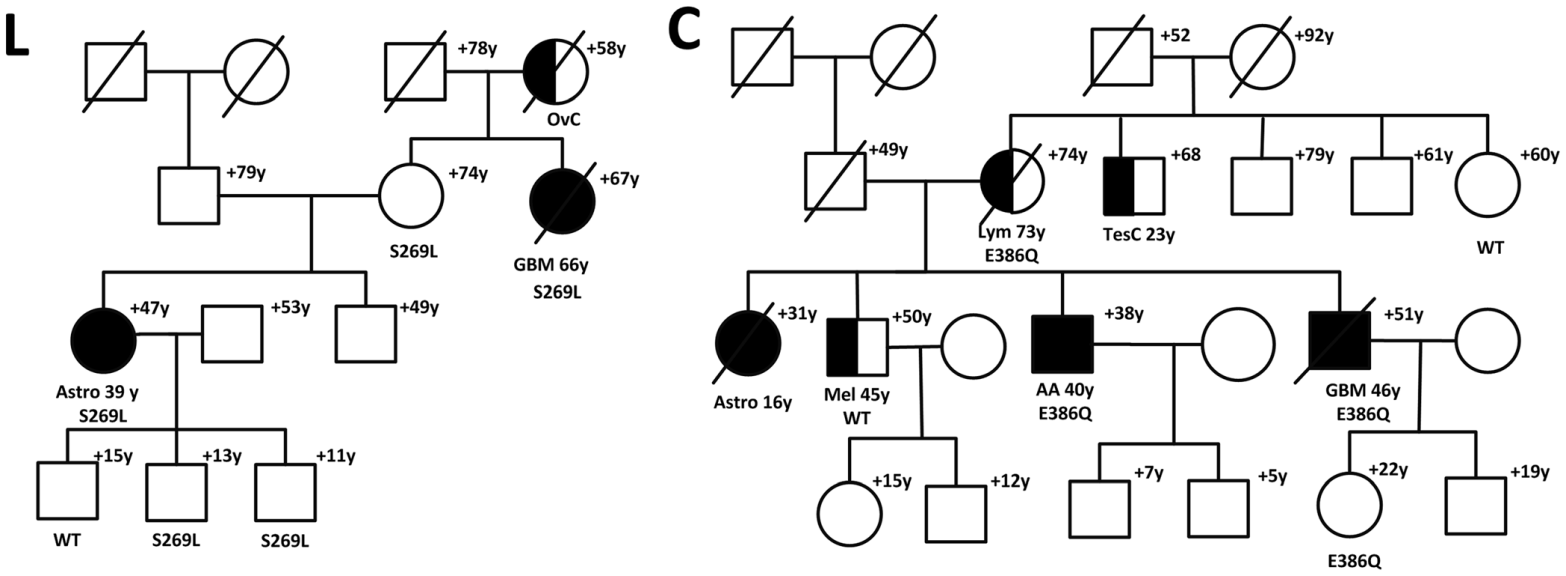
(a) *SPAG9*(L) and (b) *RUNDC1*(C) mutations in familial glioma pedigrees. Individuals with glioma are shown as filled. Individuals with other cancers are shown as half-filled. Disease and age in years (y) at first diagnosis is given underneath the symbol, current age or age at death (+) above it. Glioma type is shown (AA, anaplastic astroctyoma; Astro, astrocytoma; GBM, glioblastoma multiforme). Other cancers in the pedigree are shown (OvC, ovarian cancer; Mel, melanoma;TesC, testicular cancer; Lym, lymphoma). Carriers of *SPAG9* (a) and *RUNDC1* (b) mutations are shown with their specific mutation, whereas individuals who tested negative for the mutation in the specific pedigree are depicted as wild-type (WT).

Of the 21 candidate variants identified by our filtering schema, 19 had scaled C-scores above 10, indicating that these variants are predicted to be in the top 10% of the most deleterious possible substitutions in the human genome. The highest scaled C-scores belonged to *SPAG9* rs143491486 (C-score: 32.0), *RUNDC1* rs61995866 (C-score: 28.2), and the variant in *TTC25* (chr17:40091564 C>G; C-score: 25.9). In Family L, both sequenced affected individuals and 3 out of 4 sequenced unaffected individuals had the *SPAG9* rs143491486 variant. However, 2 of the 3 unaffected individuals who had this *SPAG9* variant were children (Fig. 3a). The abbreviated CADD output is provided as supplementary information.

## Discussion

In this study, we attempted to identify variants on targeted regions of chromosome 17q that could explain linkage across familial glioma cases. We also sought to describe the distribution of potentially deleterious rare variants within participating glioma families. As in any NGS study, we obtained a large list of variants and had to devise a filtering and verification strategy to refine our list for inclusion of only the most relevant variants (Fig. 2). Although we did not detect a variant (or gene) that could explain linkage across all participating families, we did identify several very rare or novel missense variants (many of which are predicted to be in the top 10% of the most deleterious substitutions possible in the human genome) that segregated in sequenced affected members of individual families. In complex diseases such as glioma, the importance of identifying deleterious rare variants (e.g., private mutations) should not be underestimated, as such variants may pinpoint genes that could be important to disease pathogenesis. Furthermore, despite being infrequent in the general population, the identification of these rare variants can highlight genes or genomic regions in which a series of less deleterious and more common mutations may interact to cumulatively increase disease risk in a larger population.

Two interesting candidate genes identified in this study are *MYO19* and *KIF18B*, which were the only genes that were implicated in more than one family. *MYO19* codes for a myosin that is involved in mitochondrial motility^16^. The *MYO19* gene has not been well-studied in relation to carcinogenesis; however, one study has implied that it may act as a fusion gene in breast cancer tissues^17^. *KIF18B* encodes a protein product that regulates microtubule dynamics (i.e., microtubule length) and thus, plays an important role in cell division through its involvement in mitotic spindle assembly^18^,^19^,^20^. *KIF18B* has been shown to be overexpressed in hepatocellular carcinoma, and some evidence suggests that the expression of this gene may also be deregulated in several other types of tumor tissues^20^,^21^. Both of these genes warrant additional examination in future etiologic studies.

A promising candidate variant identified in our study, *SPAG9* rs143491486 (p.Ser269Leu), is predicted to be among the top 0.1% of the most deleterious variants possible in the human genome (Table 2, Family L), based on its Combined Annotation-Dependent Depletion^22^ scaled C-score of 32. The product of this gene, sperm-associated antigen 9 (SPAG9), is a member of a scaffolding protein family that helps MAP-kinases bind with their transcription factors to activate specific signal transduction pathways^23^,^24^,^25^. SPAG9 is actually a putative oncoprotein that has been implicated in prostate, breast, hepatocellular, thyroid, cervical, lung, bladder, and endometrial cancers^23^,^24^,^26^,^27^,^28^,^29^,^30^,^31^,^32^,^33^,^34^. Recently, Yi et al. showed that *SPAG9* was differentially overexpressed in human astrocytomas, compared to normal astrocytes, and that SPAG9 expression levels were positively correlated with tumor grade (p<0.001)^24^. Interestingly, in our study, one of the two sequenced affected individuals carrying the *SPAG9* variant in Family L had a Grade II astrocytoma (Fig. 3a). Although the exact mechanism through which this particular *SPAG9* variant may be involved in gliomagenesis is unknown, it has been suggested that the SPAG9 protein may influence cell invasion by upregulating the expression of MMP9 (matrix metallopeptidase 9, a.k.a. gelatinase-B), which, in turn, has been shown to be involved in the neovascularization of malignant gliomas^35^,^36^.

The non-synonymous *SPAG9* variant identified in our study contributes to existing evidence that this gene may play an important role in glioma susceptibility. This variant is predicted to be protein damaging by SIFT (SIFT score: 0; http://sift.jcvi.org/)^37^ and PolyPhen (Polymorphism Phenotyping score: 0.997)^38^, and is at a location that is highly evolutionarily conserved. Ascertainment of whether currently unaffected members of Family L who carry this variant develop glioma in the future will be extremely important, as the unaffected individuals carrying this variant were under the age of 20 and may develop glioma later in life (Fig. 3a).

There is little known about the variant with the next highest C-score, *RUNDC1* rs61995866 (p.Glu386Gln). This variant is predicted to be in the 1% most deleterious of all possible substitutions in the human genome. The RUNDC1 protein has been shown by a high-throughput RNA interference-based screening study to be a p53 regulator^39^,^40^. Interference with this gene resulted in increased p53 transcription, thus indicating that the RUNDC1 protein may be a p53 antagonist^39^. However, this variant has not previously been studied in relation to familial glioma. Both sequenced affected individuals in Family C carried this mutation, as did two of four sequenced unaffected individuals (implying incomplete penetrance) [Fig. 3b]. However, one of the two unaffected individuals carrying the RUNDC1 variant was only in her early 20s at last contact.

Even the variants on our final list that have lower C-scores may provide interesting candidates for further investigation. For example, the candidate gene with the highest number of somatic non-silent mutations in The Cancer Genome Atlas (TCGA) low-grade glioma database is *G6PC* with three tumor samples containing missense somatic variants, followed by one tumor sample for each of *MYO19*, *GHDC* ¸ and *KRT27*. Looking at the combined low-grade glioma and glioblastoma multiforme TCGA data, *G6PC* remains the gene with the highest number of somatic non-silent mutations (four tumor samples) followed by *MYO19* (three tumor samples) and *KIF18B* (two tumor samples). This was based on an analysis of 279 low-grade glioma and 268 glioblastoma multiforme tumor samples from the TCGA project as reported by the International Cancer Genome Consortium (https://dcc.icgc.org/).

Targeted deletion of *G6PC* in mice leads to hepatic tumorigenesis^41^, and this gene is expressed at comparable levels in the central nervous system according to the Cancer Genome Anatomy Project^42^. Along with the finding that both affected (with low grade glioma) and no unaffected members of Family A carried the *G6PC* missense variant in their germline (scaled C-score: 14.5), this information makes the *G6PC* variant another interesting candidate for future study.

It is also noteworthy that four different keratin genes were implicated in our final list (Table 2, Families C, H, K, and J). Overexpression of cytokeratins has been reported in some cancers^43^,^44^,^45^, but little is known about the potential role of these genes in gliomagenesis. There is a cluster of keratin-related genes or pseudogenes on chromosome 17q,^45^ thereby obscuring whether these findings may truly be biologically relevant or simply represent an artifact of our loci of interest.

Glioma is a complex disease where multiple loci are likely to be important for disease development within a family. In order to maximize resources, the regions targeted for sequencing in our study were chosen strategically to ensure comprehensive coverage of what were likely to be the most pertinent linkage regions. However, within our sequencing targets, there are numerous regions that are notoriously difficult to sequence via the NGS platform and Sanger sequencing, possibly due to genomic duplications or high repeat content. Additionally, we acknowledge that other loci, outside of the regions covered by this study, have previously been linked to familial glioma^46^ (although no causative variants in these regions have been definitively established). For example, in our other analyses, we have found that two Gliogene families (D and R) had *TP53* mutations that may help explain their increased glioma risk^47^. Interestingly, neither of these families had chromosome 17q variants that met our final filtering criteria in the current study.

One of the challenging aspects of studying diseases with high mortality, such as glioma, is that there are very few families for which we can obtain biologic samples from more than one or two affected family members, which limits the possibility of performing segregation analyses of rare variants. Only about 5% of glioma patients have a family history of glioma^14^, and in 83% of glioma families, there are only two glioma patients in the family^11^, indicating a complex disease with low penetrance of causal variants. Unfortunately, we were unable to detect a variant that could explain linkage across all participating families in this study. Because there are no established filtering strategies for studies such as this one, we had to develop the filtering schema presented here to maximize the potential of finding such variants, while simultaneously excluding variants common among healthy populations.

Despite these limitations, the findings of this study may lend a substantive amount of insight into genes involved in gliomagenesis, and future studies should evaluate the candidate genes/regions identified here for the presence of more common/less deleterious variants that may cumulatively impact familial glioma risk in a larger population. Overall, our study complements the previous research conducted by the Gliogene Consortium on the enigmatic factors contributing to familial glioma risk. The importance of the chromosome 17q region is becoming increasingly clear, though several additional studies are necessary before definitively conclusions can be drawn. We are currently in the process of obtaining copy number variation (CNV) data for a subset of the Gliogene families. The CNV data will be examined in the context of what is currently known about factors that predict familial glioma susceptibility in the hopes that enough information can be amassed from the Gliogene Consortium's series of studies to be able to differentiate and provide genetic counseling to high-risk families and individuals.

## Methods

### Study Population

Families recruited for participation in the Gliogene Consortium had at least two biologically related members (83% of families with two and 17% with three or more affected family members) who were diagnosed with a histologically-confirmed glioma^11^. The study population and recruitment scheme have been described in detail elsewhere^11^,^13^,^14^,^15^. Briefly, for the analyses presented here, the glioma families (n = 23) contributing most to the linkage peaks identified in Shete et al. (2011)^13^ and Sun et al. (2012)^15^ were selected for genomic sequencing.

### Sample collection

Blood or saliva samples from glioma family members were obtained under written informed consent at each Gliogene Consortium recruitment center. DNA samples were prepared as previously described^13^. This study was approved by the Institutional Review Board of each Gliogene institution, including Baylor College of Medicine, and was conducted in accordance with the Declaration of Helsinki. Informed consent was obtained from all participants or their guardians.

### Library construction

DNA samples were constructed into Illumina paired-end pre-capture libraries according to the manufacturer's protocol (Illumina Multiplexing_SamplePrep_Guide_1005361_D) with modifications as described in the BCM-HGSC protocol (Illumina Barcoded Paired-End_Capture_Library_Preparation). Libraries were prepared using Beckman robotic workstations (Biomek NXp and FXp models). Briefly, 1 ug of DNA was sheared into fragments of approximately 300–400 base pairs with the Covaris E210 system followed end-repair, A-tailing, and ligation of the Illumina multiplexing PE adaptors. Pre-capture ligation-mediated PCR (LM-PCR) was performed for 6–8 cycles of amplification using the 2X SOLiD Library High Fidelity Amplification Mix (a custom product manufactured by Invitrogen). Purification was performed with Agencourt AMPure XP beads after enzymatic reactions, and following the final purification, quantification and size distribution of the pre-capture LM-PCR product was determined using the LabChip GX electrophoresis system (PerkinElmer).

### Custom capture design

The DNA capture reagent was designed to target all coding exons of all genes, the UTRs of cancer related genes, miRNA binding sites in all 3′ UTRs, transcription factor binding sites, miRNA and small nucleolar RNA within the chromosome 17 linkage peak region.

The pre-capture libraries were pooled as a 46-plex (totaling 1 ug per pool) and hybridized in solution to the custom Gliogene capture reagent (1.6 Mb, NimbleGen) according to the manufacturer's protocol (NimbleGen SeqCap EZ Exome Library SR User's Guide) with minor revisions. Human COT1 DNA was added into the hybridization to block repetitive genomic sequences, followed by post-capture LM-PCR amplification using the 2X SOLiD Library High Fidelity Amplification Mix with 14 cycles of amplification. After the final AMPure XP bead purification, quantity and size of the capture library was analyzed using the Agilent Bioanalyzer 2100 DNA Chip 7500. The efficiency of the capture was evaluated by performing a qPCR-based quality check on the enrichment level of four standard NimbleGen internal control loci. Successful enrichment of the capture libraries was estimated to range from a 6 to 9 of ΔCt value over the non-enriched samples.

### Sequencing

Library templates were prepared for sequencing using Illumina's cBot cluster generation system with TruSeq PE Cluster Generation Kits. Briefly, these libraries were denatured and diluted in hybridization buffer in order to achieve a load density of ~800 K clusters/mm^2^. Each library pool was loaded in a single lane of a HiSeq flow cell, with 2% phiX control library spiked into the lane for run quality control. The sample libraries then underwent bridge amplification to form clonal clusters, followed by hybridization with the sequencing primer. The sequencing run was performed in paired-end mode using the Illumina HiSeq 2000 platform. Using the TruSeq SBS Kits, sequencing-by-synthesis reactions were extended for 101 cycles from each end, with an additional 7 cycles for the index read. With the sequencing runs yielding an average of ~838 Mb per sample, samples achieved an average of 86% of the targeted exome bases covered to a depth of 20X or greater.

Initial sequence analysis was performed using the HGSC Mercury analysis pipeline (https://www.hgsc.bcm.edu/content/mercury)^48^. In summary, the .bcl files produced on-instrument were first transferred into the HGSC analysis infrastructure by the HiSeq Real-time Analysis module. Mercury then ran the vendor's primary analysis software (CASAVA) to de-multiplex pooled samples and generate sequence reads and base-call confidence values (qualities), followed by the mapping of reads to the GRCh37 Human reference genome (http://www.ncbi.nlm.nih.gov/projects/genome/assembly/grc/human/) using the Burrows-Wheeler aligner (BWA, http://bio-bwa.sourceforge.net/)^49^. The resulting BAM (binary alignment/map)^50^ file underwent quality recalibration using GATK (http://www.broadinstitute.org/gatk/)^51^, and where necessary the merging of separate sequence-event BAMs into a single sample-level BAM. BAM sorting, duplicate read marking, and realignment to improve insertion/deletion discovery all occur at this step.

#### Sanger validation

Mutation validation was performed with bidirectional sequencing of the selected sample sites. PCR reactions were prepared using 5 ng of genomic DNA, 0.4 µM oligonucleotide primers, and 0.7X Qiagen Multiplex Master Mix (Cat. no. 206145) containing HotStar Taq, buffer, and polymerase. Reactions were performed with and without QSOL PCR additive to enhance PCR and final sequence performance. Touchdown PCR was performed with the following parameters: 98°C for 5 min. and 10 cycles of 98°C for 30 sec., 72°C for 30 sec. (decreasing by 1°C per cycle), and 72°C for 1 min. The reaction then continued with 30 cycles of 98°C for 30 sec., 63°C for 30 sec., and 72°C for 1 min, followed by a final extension at 72°C for 5 min. The PCR products were purified with a 1:15 dilution of Exo-SAP, diluted by 0.6X, and then cycle-sequenced for 25 cycles using a 1/64^th^ dilution of BigDye Terminator v3.1 reaction mix (Applied Biosystems, Cat. No. 4337456). Finally, reactions were precipitated with ethanol, resuspended in 0.1 mM EDTA, and analyzed on AB 3730xl sequencing instruments using the Rapid36 run module and 3xx base-caller. SNPs were identified using SNP Detector software and then visually validated with Consed.

### Variant annotation and filtering

Variants were annotated for functional effect on protein and presence in variant databases (i.e., dbSNP), as well as known minor allele frequency using AnnoVar^52^. We developed a filtering schema (described below) to evaluate the full list of variants and reduce it to a subset of variants more likely to be etiologically relevant (i.e., very rare protein-altering candidate variants within the linkage region). We implemented this series of filtering strategies using scripts written in R (version 2.15.3).

Variants that met initial filtering criteria were verified by Sanger sequencing among affected individuals, and an additional post-Sanger filtering strategy was then applied. Filtering was carried out in a stepwise fashion with verification of the excluded variants at each step to ensure that biologically significant variants were not being eliminated.

#### Initial Filtering Strategy

The borders of the linkage region were defined based on a 1.7 LOD drop region (~ 99% linkage confidence interval) from the linkage peaks identified in our previous two analyses^13^,^15^, which encompassed two adjacent regions on chromosome 17q. Variants located outside this linkage region were excluded (“Linked locus” filter), unless the variants outside the region were in genes found in the Cancer Gene Census (CGC)^53^. The CGC, which is maintained by Welcome Trust Sanger Institute, is a catalogue of variants that have previously been implicated to have a causal role in oncogenesis. At the time of this analysis, the following 23 genes on chromosome 17 were included in the CGC: *TAF15, SUZ12, LASP1, CLTC, CDK12, NF1, RARA, BRCA1, DDX5, ETV4, MLLT6, ERBB2, COL1A1, BRIP1, HLF, CD79B, MSI2, PRKAR1A, MSF, ASPSCR1, CANT1, SRSF2,* and *ALO17*.

Among non-CGC genes, variants that were not within 200 bases of the sequencing target regions were excluded (“Targeted Region” filter). Next, common variants, defined as those that had >5% frequency in the Exome Sequencing Project (ESP5400 release)^54^ or 1000 Genome (1000G, 2010_07 release)^55^ CEU population, were also excluded (“Rare Variant” filter). Finally, we removed any variant that was not shared by all sequenced affected individuals of at least one family, prior to Sanger verification (“Family Segregation” filter).

#### Post-Sanger Filtering Strategy

Variants were considered verified if Sanger reads had full concordance with NGS reads in all affected individuals, and both the forward and reverse reads agreed. Final filtering involved excluding the following: variants outside of the linkage region (“Linked locus” filter); variants with a minor allele frequency >0.1% in the ESP6500 release or 1000G 2012_04 release (“Very Rare Variant” filter); variants not within the exonic sequencing target regions (“Targeted Exome” filter); and variants that did not alter the amino acid sequence of the product protein (“Protein Alteration” filter). The aim of this filtering strategy was to narrow down the list of variants to a list of very rare protein-altering candidate variants within our linkage locus. This was, however, carried out in a step-wise fashion with verification of the excluded variants at each step for potential biological function to ensure we are not excluding any biologically significant variants.

#### Combined Annotation-Dependent Depletion C-Score

After implementing the above filtering schema, we used scaled C-scores from the Combined Annotation-Dependent Depletion (CADD) method to rank the variants remaining on our final list by potential deleteriousness^22^. The CADD C-score provides an integration of 63 different annotations (i.e., allelic diversity, functionality, putative pathogenicity, and evolutionary conservation) into one score per variant. For example, the C-score integrates scores from PolyPhen^38^ and SIFT^37^ for predicted impact of the variant on protein function, GERP^56^ and PhyloP^57^ for evolutionary conservation, and a number of other annotations for prioritization of causal variants^22^. A scaled C-score ≥10 indicates variants predicted to be in the top 10% of the most deleterious possible substitutions in the human genome, a score of ≥20 indicates variants predicted to be in the top 1%, and a score of ≥30 indicates variants predicted to be in the top 0.1%. More details on the CADD C-score can be found elsewhere^22^.

### Ethics

Written informed consent was obtained from each subject or from his or her guardian. Approval from local institutional review boards was received at each Gliogene participating institution. This study was conducted in accordance with the Declaration of Helsinki.

## Author Contributions

A.J. and E.S.A. wrote the main text of the manuscript with significant input from M.N.A., S.N.J., G.N.A., J.S.B.S., C.I.A., D.M.M., B.S.M. and M.L.B. A.J. prepared figures 1-2, and G.N.A. prepared figure 3. A.J., E.S.A., M.N.B., G.N.A., Y.L., S.T., S.E.P., S.S., C.I.A., B.S.M. and M.L.B. contributed to the analysis with substantial input from all other authors. All authors reviewed the manuscript and were involved in study design and conduct.

## Supplementary Material

Supplementary Information Targeted Sequencing in Chromosome 17q Full CADD Output

## Figures and Tables

**Table 1:** Characteristics of and relationships between individuals affected by histologically-confirmed glioma in each family

Relationship	Histology	Age Dx	Sex	Sequenced
Family A				
Proband	Oliogdendroglioma	48	Female	Yes
Sibling	Astrocytoma	33	Male	Yes
P 2nd Cousin	Ependymoma	20	Female	No
Family B				
Proband	Astrocytoma	40	Female	Yes
P 1st Cousin	Oliogendrglioma	30	Female	Yes
Family C				
Proband	Anaplastic Astro	40	Male	Yes
Sibling	GBM	46	Male	Yes
Sibling	Astrocytoma	16	Female	No
Family D				
Proband	GBM	54	Female	Yes
Father	UNK Glioma	31	Male	No
P Aunt	GBM	55	Female	No
Family E				
Proband	Anaplastic Astro	39	Male	Yes
Sibling	Oligodendroglioma	27	Female	Yes
Family F				
Proband	GBM	61	Male	Yes
Sibling	GBM	52	Female	Yes
Family G				
Proband	Anaplastic Oligo	28	Male	Yes
Father	Anaplastic Astro	29	Male	Yes
P Grandfather	UNK Glioma	50	Male	No
Family H				
Proband	GBM	59	Male	Yes
Sibling	UNK Glioma	42	Male	No
Family I				
Proband	Anaplastic Oligo	58	Female	Yes
Nephew	Oligodendroglioma	32	Male	Yes
Family J				
Proband	Anaplastic Astro	27	Female	Yes
Sibling	Oligoastrocytoma	38	Male	Yes
Family K				
Proband	Anaplastic Oligo	65	Male	No
M Aunt	GBM	82	Female	Yes
M Great Aunt	GBM	79	Female	No
M Great Aunt	Anaplastic Astro	81	Female	No
M 1st Cousin Once Removed	UNK Glioma	63	Female	No
M 1st Cousin Once Removed	GBM	61	Male	No
Family L				
Proband	Astrocytoma	39	Female	Yes
M Aunt	GBM	66	Female	Yes
Family M				
Proband	GBM	66	Female	Yes
M Aunt	GBM	59	Female	No
Family N				
Proband	GBM	66	Male	Yes
Sibling	GBM	53	Male	No
Family O				
Proband	Astrocytoma	43	Female	Yes
Mother	UNK Glioma	27	Female	No
Sibling	GBM	45	Male	No
Family P				
Proband	Anaplastic Astro	28	Male	Yes
Identical Twin	Oligoastrocytoma	18	Male	Yes
Family Q				
Proband	GBM	10	Female	Yes
M Grandmother	GBM	65	Female	No
Family R				
Proband	GBM	44	Female	Yes
Sibling	GBM	22	Male	No
Niece	Astrocytoma	24	Female	Yes
Niece	Astrocytoma	4	Female	No
M Aunt	UNK Glioma	11	Female	No
M 1st Cousin	Astrocytoma	38	Female	Yes
M 2nd Cousin Once Removed	UNK Glioma	49	Female	No
M 2nd Cousin Once Removed	UNK Glioma	46	Female	No
P 1st Cousin Twice Removed	UNK Glioma	78	Male	No
P 2nd Cousin Once Removed	UNK Glioma	4	Male	No
Family S				
Proband	Oligodendroglioma	37	Female	Yes
Sibling	Ana Oligoastro	41	Male	Yes
Family T				
Proband	GBM	68	Female	Yes
Mother	GBM	78	Female	No
M Uncle	UNK Glioma	49	Male	No

P, Paternal; M, Maternal; Dx, Diagnosis; UNK, unknown; Ana, anaplastic; GBM, glioblastoma multiforme; Astro, astrocytoma.

**Table 2:** List of 21 very rare or novel missense variants within the linkage locus that segregate among all affected members of individual families. Listed in order of the Combined Annotation-Dependent Depletion scaled C-scores^22^, each variant is private to one family. Families not listed had no variants that survived filtration. The affected (or unaffected) ratio denotes the number of sequenced affected (or unaffected) individuals carrying the variant over total number of sequenced affected (or unaffected) individuals within the family

FamilyID	Chr. 17 Position	Ref. Allele	Var. Allele	Gene Symbol	ESP6500 Freq.	1000GFreq.	dbSNP ID	AffectedRatio	Unaffected Ratio	Scaled C-Score
L	49098662	G	A	*SPAG9*	0.069%		rs143491486	2/2	3/4	32.0
C	41143047	G	C	*RUNDC1*	0.069%	0.05%	rs61995866	2/2	2/4	28.2
M	40091564	C	G	*TTC25*				1/1	5/8	25.9
O	34883425	G	A	*MYO19*				1/1	4/5	19.2
C	39503458	C	T	*KRT33A*	0.015%		rs142400197	2/2	1/4	19.2
G	43005646	G	C	*KIF18B*	0.090%		rs202002436	2/2	0/4	18.8
B	43006370	C	T	*KIF18B*	0.008%		rs201865018	2/2	1/6	18.4
P	40345030	C	A	*GHDC*	0.038%	0.09%	rs149568450	2/2	2/4	18.1
J	34859014	C	T	*MYO19*				2/2	3/8	17.4
P	42750898	A	T	*C17orf104*		0.05%	rs192757598	2/2	2/4	16.2
A	41063291	G	A	*G6PC*				2/2	0/5	14.5
L	37785802	C	A	*PPP1R1B*			rs201594054	2/2	2/4	14.1
H	38933291	G	A	*KRT27*	0.077%		rs148928902	1/1	2/6	14.0
C	45904542	C	T	*MRPL10*	0.046%	0.05%	rs149631185	2/2	3/4	14.0
J	48561815	T	C	*RSAD1*				2/2	3/8	12.2
S	41338453	G	C	*NBR1*	0.042%		rs200709037	2/2	3/6	11.3
B	48141461	G	A	*ITGA3*		0.05%	rs201210478	2/2	1/6	10.7
K	39036435	C	T	*KRT20*				1/1	0/24	10.5
J	39620632	G	A	*KRT32*	0.008%		rs147094229	2/2	4/8	7.2
K	55194252	G	A	*AKAP1*	0.062%		rs149147838	1/1	1/24	6.7
N	45419305	C	G	*EFCAB13*	0.038%		rs138179179	1/1	4/7	5.0
