# Supplementary material for: Targeted Sequencing in Chromosome 17q Linkage Region Identifies Familial Glioma Candidates in the Gliogene Consortium
Source: Sci Rep. 2015 Feb 5;5:8278. doi: 10.1038/srep08278 (PMC4317686; doi:10.1038/srep08278)
Supplement: Supplementary Information — Targeted Sequencing in Chromosome 17q Full CADD Output [file srep08278-s1.pdf]

Targeted Sequencing in Chromosome 17q Linkage Region Identifies Familial Glioma Candidates in the Gliogene Consortium

Ali Jalali, E. Susan Amirian, Matthew N. Bainbridge, Georgina N. Armstrong, Yanhong Liu, Spyros Tsavachidis, Shalini N. Jhangiani, Sharon E. Plon, Francis Ali-Osman, Siegal Sadetzki, Christoffer Johansen, Richard S. Houlston, Robert B. Jenkins, Daniel Lachance, Sara H. Olson, Ching C. Lau, Elizabeth B. Claus,
Jill S. Barnholtz-Sloan, Dora Il'yasova, Joellen Schildkraut, Jonine L. Bernstein, Ryan T. Merrell, Margaret R. Wrensch, Faith G. Davis, Rose Lai, Sanjay Shete, Kenneth Aldape, Christopher I. Amos, The Gliogene Consortium, Donna M. Muzny, Richard A. Gibbs, Beatrice S. Melin, and Melissa L. Bondy

CADD v1.0 (c) University of Washington and Hudson-Alpha Institute for Biotechnology 2013. All rights reserved.

#Chrom	Pos	Ref	Anc	Alt	Type	Consequence	ConsScore	ConsDetail	GC	CpG	priPhCons	mamPhCons	verPhCons	priPhyloP	mamPhyloP	verPhyloP	GerpN	GerpS	GerpRS	GerpRSpva	GeneName	cDNApos	CDSpos	protPos	Exon	Grantham	PolyPhenCat	PolyPhenVal	SIFTcat	SIFTval	RawScore	Cscore
17	49098662	G	G	A	SNV	NON_SYNONYMOUS	7	missense	0.42	0.04	0.998	0.999	1	0.559	2.713	4.464	5.74	5.74	721.7	3.06E-87	SPAG9	637	419	140	4-Apr	145	probably_damaging	0.997	deleterious	0	5.105154	32
17	41143047	G	G	C	SNV	NON_SYNONYMOUS	7	missense	0.58	0	0.997	1	1	0.651	2.715	6.001	5.24	5.24	2562.4	9.36E-271	RUNDC1	1168	1156	386	5-May	29	possibly_damaging	0.815	deleterious	0.01	4.895174	28.2
17	40091564	C	C	G	SNV	NON_SYNONYMOUS	7	missense	0.56	0.04	0.908	1	1	0.651	2.752	3.149	5.82	4.66	501.6	1.44E-68	AC091172.1	288	209	70	8-Feb	60	possibly_damaging	0.736	deleterious	0.01	4.688063	25.9
17	34883425	G	G	A	SNV	NON_SYNONYMOUS	7	missense	0.6	0.05	0.884	0.331	0.741	-0.155	0.941	1.345	5.35	2.11	1249.3	8.05E-100	MYO19	1270	257	86	26-May	145	possibly_damaging	0.734	deleterious	0.04	3.786501	19.22
17	39503458	C	C	T	SNV	NON_SYNONYMOUS	7	missense	0.52	0.05	0.984	0.999	1	0.651	2.544	5.209	4.6	4.6	882.3	7.67E-70	KRT33A	650	605	202	7-Apr	29	benign	0.13	deleterious	0.01	3.783172	19.21
17	43005646	G	G	C	SNV	NON_SYNONYMOUS	7	missense	0.64	0.05	0.869	0.991	0.997	0.557	2.354	3.711	4.54	4.54	793	1.68E-38	KIF18B	2076	2060	687	14-Dec	103	probably_damaging	0.999	deleterious	0	3.705459	18.82
17	43006370	C	G	T	SNV	NON_SYNONYMOUS	7	missense	0.6	0.05	0.382	0.997	0.743	0.557	1.414	0.785	5.17	3.1	319.7	5.44E-34	KIF18B	1592	1576	526	14-Nov	29	benign	0.007	deleterious	0.03	3.619369	18.42
17	40345030	C	C	A	SNV	NON_SYNONYMOUS	7	missense	0.56	0.04	0.981	1	1	0.557	1.235	1.509	4.77	3.8	166.3	1.90E-12	GHDC	518	281	94	10-Apr	102	probably_damaging	0.994	tolerated	0.15	3.548857	18.1
17	34859014	C	C	T	SNV	NON_SYNONYMOUS	7	missense	0.51	0.04	0.027	0.507	0.672	0.559	2.611	3.164	5.25	5.25	126.5	9.64E-13	MYO19	3016	2003	668	21/26	43	probably_damaging	1	deleterious	0	3.36766	17.35
17	42750898	A	A	T	SNV	NON_SYNONYMOUS	7	missense	0.35	0.03	0.893	1	0.999	-0.302	0.442	0.143	5.67	3.46	934.4	4.79E-175	C17orf104	2764	2622	874	8-Jul	110	possibly_damaging	0.556	deleterious	0.04	3.054785	16.2
17	41063291	G	G	A	SNV	NON_SYNONYMOUS	7	missense	0.56	0.08	0.972	0.991	0.983	-0.163	0.278	0.802	5.07	1.92	1300.6	3.93E-115	G6PC	1001	922	308	5-May	29	benign	0.011	deleterious	0.03	2.561783	14.53
17	37785802	C	C	A	SNV	NON_SYNONYMOUS	7	missense,N	0.63	0.04	0.978	1	1	0.557	2.476	3.035	5	5	123.3	3.41E-15	PPP1R1B	243	152	51	7-Mar	126	NA	NA	NA	NA	2.425582	14.07
17	38933291	G	G	A	SNV	NON_SYNONYMOUS	7	missense	0.45	0.01	0.959	0.981	0.991	0.645	1.476	1.958	5.66	4.67	389.4	2.17E-34	KRT27	1381	1340	447	8-Aug	89	benign	0.022	tolerated	0.07	2.404546	14
17	45904542	C	C	T	SNV	NON_SYNONYMOUS	7	missense	0.53	0.05	0.634	0.747	0.662	-0.298	0.31	0.879	5.1	1.98	451.3	6.45E-36	MRPL10	734	281	94	5-Mar	43	benign	0.08	tolerated	0.07	2.395683	13.97
17	48561815	T	T	C	SNV	NON_SYNONYMOUS	7	missense,N	0.64	0.03	0.829	0.999	0.999	0.53	2.184	2.819	5.76	5.76	336.2	2.88E-40	RSAD1	584	584	195	6-May	64	benign	0.031	NA	NA	1.874167	12.23
17	41338453	G	G	C	SNV	NON_SYNONYMOUS	7	missense	0.44	0.01	0.663	0.944	0.253	0.604	0.022	-0.093	5.43	-0.761	500.5	6.01E-43	NBR1	853	394	132	21-Jun	27	benign	0.003	tolerated	0.13	1.5897	11.27
17	48141461	G	G	A	SNV	NON_SYNONYMOUS	7	missense	0.66	0.08	0.996	0.996	0.995	0.651	-0.007	0.177	5.27	-0.287	418.9	1.08E-51	ITGA3	557	227	76	26-Feb	43	benign	0.004	tolerated	0.83	1.429968	10.72
17	39036435	C	C	T	SNV	NON_SYNONYMOUS	7	missense	0.5	0.03	0.513	0.001	0	0.486	-0.11	-0.719	5.29	-1.67	350.6	2.60E-28	KRT20	751	709	237	8-Apr	29	benign	0.004	deleterious	0.04	1.372279	10.52
17	39620632	G	G	A	SNV	NON_SYNONYMOUS	7	missense	0.64	0.13	0.944	0.353	0.456	0.651	2.544	2.808	5.13	5.13	897.7	5.04E-72	KRT32	876	772	258	7-Apr	74	benign	0.179	tolerated	0.84	0.600713	7.238
17	55194252	G	G	A	SNV	NON_SYNONYMOUS	7	missense	0.53	0.01	0.921	1	1	-0.298	1.206	2.733	5.27	4.3	289.8	5.54E-54	AKAP1	161	163	55	6-Feb	58	benign	0.015	tolerated	1	0.492639	6.672
17	45419305	C	C	G	SNV	NON_SYNONYMOUS	7	missense	0.29	0	0.01	0	0.001	-0.235	-0.161	-0.157	3.21	-1.02	NA	NA	C17orf57	577	210	70	22-Jun	10	benign	0	tolerated	0.08	0.179764	4.968
